# Persistent Abdominal Pain and Diarrhea After Appendectomy—Crohn's Disease Versus Intestinal Tuberculosis

**DOI:** 10.1002/jgh3.70157

**Published:** 2025-04-15

**Authors:** Jonas Wizenty, Martin Maibier, Michael Sigal

**Affiliations:** ^1^ Department of Hepatology and Gastroenterology Charité—Universitätsmedizin Berlin Berlin Germany; ^2^ Berlin Institute of Health at Charité—Universitätsmedizin Berlin, BIH Biomedical Innovation Academy, BIH Charité Clinician Scientist Program Berlin Germany; ^3^ Berlin Institute for Medical Systems Biology (BIMSB), Max Delbrück Center for Molecular Medicine in the Helmholtz Association (MDC) Berlin Germany

**Keywords:** gastrointestinal infection, IBD, intestinal tuberculosis

## Abstract

**Case Presentation:**

In Western Europe, intestinal tuberculosis is a rare differential diagnosis for Crohn's disease. In this report, we present a case of intestinal tuberculosis in a 59‐year‐old female initially suspected of Crohn's disease with persistent abdominal pain and diarrhea after appendectomy.

**Conclusion:**

This case highlights the need for TB culture in patients with positive IGRA and suspected Crohn's disease.

## 1 Case Report

**FIGURE 1:**
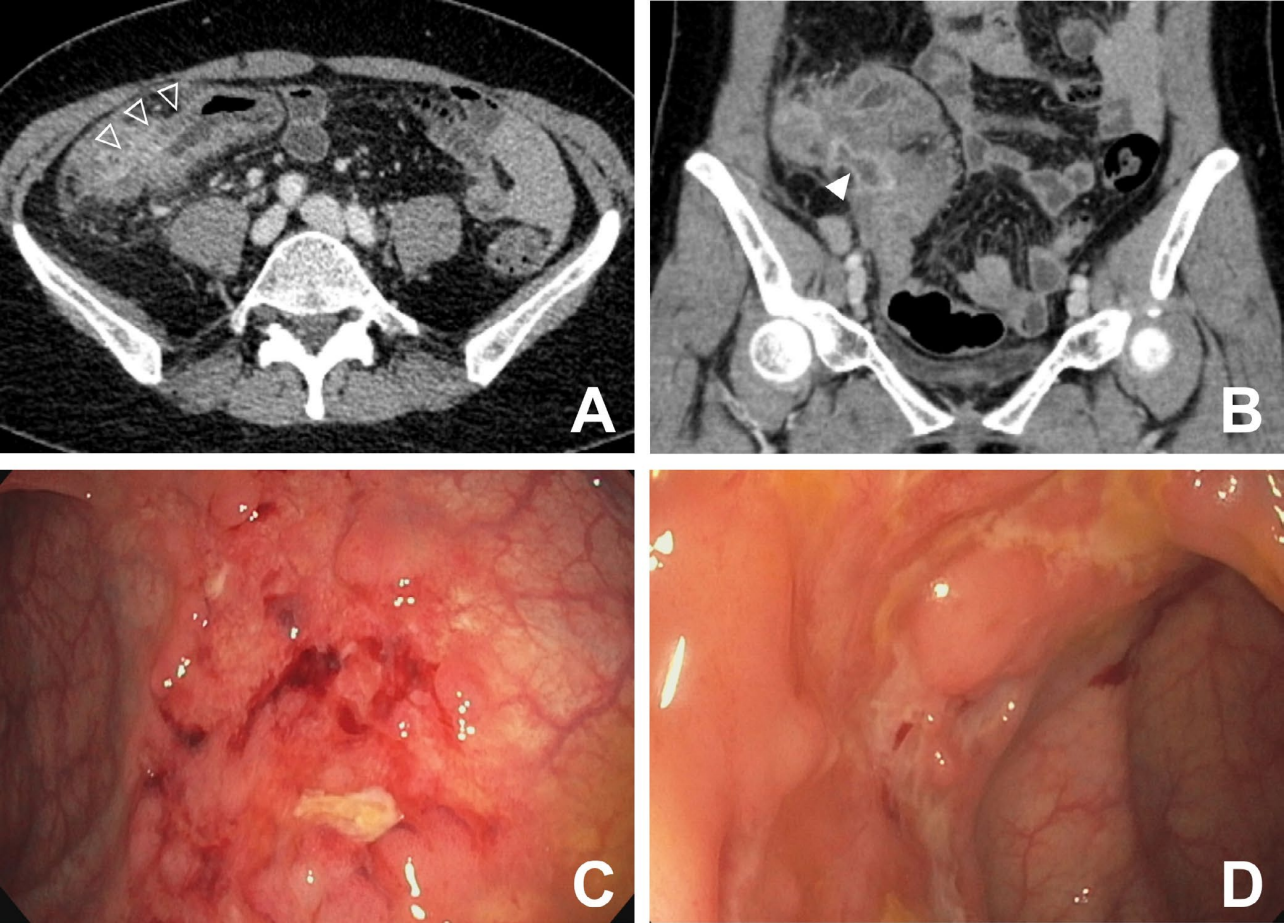
(A) Axial CT scan of the abdomen with inflammatory changes in the cecum and terminal ileum with wall thickening (empty arrowheads), perifocal fatty tissue imbibition, and locally enhancing peritoneum. (B) Coronary CT scan of the abdomen with abscess‐suspect formation (solid arrowhead) in the right lower abdomen caudal to the cecal pole and adjacent to the terminal ileum. (C, D) Endoscopic view of the cecum with ulcerating inflammation.

A 59‐year‐old female native Filipino and former healthcare professional, who moved to Europe 7 years prior, received surgical and antibiotic treatment for acute appendicitis with peritonitis. Histopathologic examination of the appendectomy specimen revealed epithelioid‐granulomatous inflammation, negative *Mycobacterium tuberculosis* (TB) complex PCR, but suspected parasite infection (i.e., *Schistosoma* ). TB culture was not performed. Following positive tests for *Clostridioides difficile* (stool), *Ascaris* (serology titer of 10 AI), and *Enterobius* (urine), the patient was treated with vancomycin, praziquantel, ivermectin, and albendazole. Despite this, the patient suffered from persistent abdominal pain, diarrhea, and weight loss. Laboratory findings revealed: hemoglobin 11 g/dL, platelets 440/nL, and C reactive protein 77 mg/L. CT imaging showed ileocecal inflammation with an abscess‐suspect formation (Figure 1A,B). Interferon‐gamma‐release assay (IGRA) was positive (TB1: 4.16 IU/mL, TB2: 3.50 IU/mL). Colonoscopy, performed 2 months after appendectomy, confirmed discontinuous ulcerating ileocecal inflammation (Figure 1C,D), and ileocecal biopsies yielded noncaseating granulomas, again negative for TB complex PCR. Based on these findings, Crohn's disease (CD) was suspected. Again, TB culture was not performed.

Given the patient's history, active TB is a relevant differential diagnosis to CD in this case. The risk of progression from latent TB infection (LTBI) to active TB disease increases strongly between interferon‐gamma levels of 0 and 5 IU/mL [1]. With two histologic specimens negative for TB, this case could have been misinterpreted as CD with concomitant LTBI [2]. However, a second colonoscopy with multiple native biopsies confirmed intestinal TB via culture and PCR. Of note, stool samples did not show any acid‐fast bacteria, and histology remained negative for TB a third time.

## 2 Discussion

In Western Europe, intestinal TB is a rare condition and difficult to distinguish from CD [3]. As therapy principles differ tremendously between CD and TB, gastroenterologists must be vigilant in interpreting IGRA results and consider native tissue sampling for culture and PCR to identify patients with intestinal TB before treating eventual CD with LTBI. Our patient rapidly recovered upon standard TB treatment.

## Conflicts of Interest

J.W. and M.M. have served as advisory board members for Janssen‐Cilag GmbH. M.S. declares no conflicts of interest.
